# Statistical Process Control of a Kalman Filter Model

**DOI:** 10.3390/s141018053

**Published:** 2014-09-26

**Authors:** Sonja Gamse, Fereydoun Nobakht-Ersi, Mohammad A. Sharifi

**Affiliations:** 1 Unit for Surveying and Geoinformation, University of Innsbruck, Technikerstr. 13, Innsbruck 6020, Austria; 2 Department of Applied Mathematics, University of Tabriz, 29 Bahman Blvd, 5166616471 Tabriz, Iran; E-Mail: fndrl361@gmail.com; 3 Department of Geomatic and Surveying Engineering, College of Engineering, University of Tehran, 111554563 Tehran, Iran; E-Mail: sharifi@ut.ac.ir

**Keywords:** consistency check, controllability, Kalman filter, measurement innovation, observability, system state

## Abstract

For the evaluation of measurement data, different functional and stochastic models can be used. In the case of time series, a Kalman filtering (KF) algorithm can be implemented. In this case, a very well-known stochastic model, which includes statistical tests in the domain of measurements and in the system state domain, is used. Because the output results depend strongly on input model parameters and the normal distribution of residuals is not always fulfilled, it is very important to perform all possible tests on output results. In this contribution, we give a detailed description of the evaluation of the Kalman filter model. We describe indicators of inner confidence, such as controllability and observability, the determinant of state transition matrix and observing the properties of the *a posteriori* system state covariance matrix and the properties of the Kalman gain matrix. The statistical tests include the convergence of standard deviations of the system state components and normal distribution beside standard tests. Especially, computing controllability and observability matrices and controlling the normal distribution of residuals are not the standard procedures in the implementation of KF. Practical implementation is done on geodetic kinematic observations.

## 1. Introduction

A key part of the process of modeling any processes is the evaluation of whether the mathematical model is accurately chosen. This is often quite difficult to assess. Models are influenced by various sources of uncertainty—such as model noise, imprecision, incompleteness, ambiguity, vagueness and, maybe most important, the lack of complete knowledge and the influence of mathematical over-simplification. Therefore, deterministic mathematical modeling of dynamic systems is usually imperfect, and a statistical approach is necessary to estimate unknown parameters and to evaluate their accuracies. In the modeling of kinematic processes, we must deal with time series of observations and standard techniques of the noise reduction of time series, including filtering and smoothing. By applying least squares estimation in the sequential mode, the removal of noise from a time series has been optimized using the well-known algorithm of Kalman filtering (KF). The algorithm is described in several books [1-6], and implemented in many non-engineering and, especially, engineering tasks [7,8], as well as many others. KF overcomes the problem of uniformly-defined unknown parameters, ensures computational integrity when dealing with redundant observations, minimizes the influence of measurement errors on system state estimation and allows one to compute the accuracy of their estimate.

After formalizing a functional model as a mathematical relationship between the measurements and unknown parameters, a reliable stochastic model is required for the propagation of the observation of random errors into the estimated parameters. In addition, systematic effects or inconsistencies in the data, the removal or appropriate down-weighting of outliers also need to be considered. In the case of KF modeling, very well-known hypothesis testing and identification of significant inconsistencies is performed in the domain of measurements and in the system state domain.

In our contribution, we describe additional, theoretically known, procedures and parameters, which can be used for the evaluation of the functional model and are not used as standards in the evaluation of KF models. We describe:

(1) indicators of inner confidence:
  ‐ controllability and observability;
  ‐ determinant of state transition matrix;
  ‐ properties of the *a posteriori* system state covariance matrix;
  ‐ properties of the Kalman gain matrix;
(2) statistical tests:
  ‐ standard deviations of the system state components;
  ‐ test statistics in the domain of the measurements;
  ‐ test statistics for the system state domain.

As a practical example, we use a reference frame as an additional estimator parameter of the theoretical background.

The paper is organized as follows. In Section 2, we describe the theoretical background for a statistical analysis of kinematic and dynamic processes. Indicators of inner confidence and the parameters for robust statistical tests are given in detail. In Section 3, we illustrate an example of the practical implementation of this theory. In Section 4, the results of the practical example are evaluated. Results, the contribution of the work and suggestions for further work are discussed in Section 5, which concludes the paper.

Some of the material covered in this contribution are closely related to the content in [9,10], where more detailed explanations of the practical example presented here, especially the development of the model and measurement system, can be found. The main intent of this contribution is to describe in detail the parameters that enable evaluation of a Kalman filter model in real-time and when no reference frame is available and no additional measurements are performed.

## 2. Statistical Process Control of Kinematic and Dynamic Processes

Evaluation models are always approximations that require robust evaluation of derived results to assess accuracy. To minimize model uncertainties and measurement errors, redundant measurements are usually made to check the system. On the other hand, statistical process control, which is based on the outputs of the process and their measurable attributes, is included into the mathematical model and helps the engineer to track and measure the variability of the process to be controlled [11].

In the case of KF, we estimate the state of a dynamic system, which evolves through time and can be modeled using a stochastic equation. KF modeling provides indicators that enable the evaluation of estimated outputs. Because these values are computed through the KF process, they can be only used as indicators of inner confidence. Consistency checks of the model must then be performed using additional statistical methods and parameters and must be addressed as more global tests.

### 2.1. Indicators of Inner Confidence

#### 2.1.1. Controllability and Observability

“The concepts of controllability and observability are fundamental to modern control theory. These concepts define how well we can control a system, *i.e.*, derive the state to a desired value, and how well we can observe a system, *i.e.*, determine the initial conditions after measuring the estimate outputs” [6] (p. 38).

#### 2.1.2. Controllability

“A discrete-time (deterministic) system is completely controllable if, given an arbitrary destination point in the state space, there is an input sequence that will bring the system from any initial state to this point by a finite number of steps” [1] (p. 30). For the *n*-state discrete linear time-invariant (for a time-invariant system, using fixed time intervals, the matrices F, G and H are independent of the discrete-time index [4]) system, the controllability condition is that the pair F, G is controllable; that is, the controllability matrix [1]:

(1) $$\text{C}=[\text{G}\;\text{F}\;\cdot \;\text{G}\;{\text{F}}^{2}\cdot \;\text{G}\;\ldots {\text{F}}^{\left(n-1\right)}\;\cdot \;\text{G}]$$

has a full rank *n*. Matrix **F** is a state-transition matrix, and matrix **G** is a matrix that relates to the optional control input. The controllability condition states that process noise enters into each state component and prevents the covariance of the state from converging to zero. This condition causes the covariance to be positive definite, *i.e.*, all of the eigenvalues are positive [1].

#### 2.1.3. Observability

A deterministic system is completely observable if its initial state can be fully and uniquely recovered from a finite number of observations of its output estimates with the given knowledge of associated inputs. For the *n*-state discrete linear time-invariant system, the observability condition is that the pair F, H is observable; that is, the observability matrix [1]:

(2) $$\text{O}={\left[\text{H}\;\text{H}\;\cdot \text{F}\;\text{H}\;\cdot {\text{F}}^{2}\ldots \text{H}\;\cdot {\text{F}}^{\left(n-1\right)}\right]}^{T}$$

has a full rank *n*. Matrix H is defined in Section 2.2.2. The observability condition on the state guarantees a steady flow of information about each state component; this prevents the uncertainty from becoming unbounded. This condition yields the existence of a (not necessarily unique) steady-state solution for the covariance matrix that is positive-definite or positive-semidefinite, *i.e.*, with finite positive or nonnegative eigenvalues, respectively [1].

“Computation of the observability matrix is subject to model truncation errors and round-off errors, which could make the difference between a singular and non-singular result. Even if the computed observability matrix is close to being singular, this is cause for concern. One should consider a system as poorly observable if its observability matrix is close to being singular. For that purpose, one can use the singular-value decomposition or the condition number of the observability matrix to define a more quantitative measure of unobservability” [4] (p. 44). The condition number of a matrix O is denned as the ratio of the largest singular value *s_max_* to the smallest *s_min_* of matrix O:

(3) $$\text{cond}(\text{O})=\frac{s_{\text{max}}}{s_{\text{min}}}\geq 1$$

“The reciprocal of its condition number measures how close the system is to being unobservable” [4] (p. 44).

#### 2.1.4. Determinant of State Transition Matrix

In general, the determinant provides important information about the design matrix of a linear system. The system has a unique solution only when the determinant is non-zero; when the determinant is zero, there are either no solutions or many solutions. In the case of KF, we asses and monitor the determinant value of the state transition matrix F*_k_*, which defines the transition from the previous state to the current system state.

#### 2.1.5. Properties of the A *Posteriori* System State Covariance Matrix

Properties of the *a posteriori* system state covariance matrix ${\text{P}}_{k}^{+}$ are generally used to obtain preliminary characteristics of the reliability of a KF model. The importance of the convergence of the trace of system state covariance matrix lies in its definition: $\text{trace}({\text{P}}_{k}^{+})$ is the sum of the diagonal elements of the matrix ${\text{P}}_{k}^{+}$, which represent variances (squares of standard deviations) of the system-state components. If the sum of diagonal elements converges towards some value, then we reason that single elements also converge towards some solution [10].

##### Eigenvalues of the *A Posteriori* System State Covariance Matrix

The solution of covariance matrix **P***_k_* should theoretically always be a symmetric-positive semi-definite (non-negative definite) matrix. An important caveat to keep in mind is that the two diagnostic properties of a covariance matrix (symmetry and positive definiteness) can be lost due to round-off errors in the course of calculating its propagation equations [1].

##### Condition Number of the *A Posteriori* System State Covariance Matrix

Mathematically, matrix ${\text{P}}_{k}^{+}$ is non-singular. However, numerical problems sometimes make matrix ${\text{P}}_{k}^{+}$ indefinite or non-symmetric. Numerical problem can, for example, arise in cases in which some elements of the state-vector x are estimated to a much greater precision than other elements of x. This could arise, e.g., because of discrepancies in the units of the state vector [6]. The discrepancy can be identified using different eigenvalues of the matrix ${\text{P}}_{k}^{+}$, specially those reflecting the condition number of matrix ${\text{P}}_{k}^{+}$, defined in [1] as the common logarithm of the ratio of its largest to smallest eigenvalue:

(4) $$\kappa ({\text{P}}_{k}^{+})={\text{log}}_{10}\frac{\lambda_{\text{max}}}{\lambda_{\text{min}}}$$

The value of the condition number depends strongly on the value of the process noise intensity scalar *σ_ω_*. The condition number can therefore reflect the ratio between process and measurement noise.

#### 2.1.6. Properties of the Kalman Gain Matrix

The inclusion of measurements into system state estimates are determined by Kalman gain K, which is influenced by the uncertainty in *a priori* state estimates and uncertainty in measurement estimates. Intuitively, measurements with high precision should result in precise state estimates. Input measurements define the system state, directly or indirectly, and thus, if they have high precision, the KF can rely on them as good indicators of the system state. The Kalman gain matrix should reflect this.

##### Intuitive Interpretation of the Gain

Taking a simplistic scalar view of the optimal Kalman gain matrix, ${\text{K}}_{k}={\text{P}}_{k}^{-}\cdot {\text{H}}_{k}^{T}\cdot {\left({\text{R}}_{k}+{\text{H}}_{k}\cdot {\text{P}}_{k}^{-}\cdot {\text{H}}_{k}^{T}\right)}^{-1}$, it is [1]:

(1) proportional to the state prediction variance, ${\text{P}}_{k}^{-}$,
(2) inversely proportional to the innovation variance, ${\text{D}}_{k}={\text{R}}_{k}+{\text{H}}_{k}\cdot {\text{P}}_{k}^{-}\cdot {\text{H}}_{k}^{T}$.

According to Bar-Shalom *et al.*, “the gain is:

(1) large if the state prediction is inaccurate (has a large variance) and the measurement is accurate (has a relatively small variance),
(2) small if the state prediction is accurate (has small variance) and the measurement is inaccurate (has a relatively large variance)” [1] (p. 207).

A large gain indicates a rapid response of the input measurement in updating the state, while a small gain yields a slower response to that measurement [1].

### 2.2. Statistical Tests

Equations for the estimation of measurement prediction covariance, innovation covariance, Kalman gain, innovation or measurement residual, updated state estimate and its updated covariance all yield filter-calculated covariances, which are exact if all of the modeling assumptions used in the filter derivation are all perfect. In practice, this is not the case, and the validity of the accuracy of these filter estimates has to be tested [1].

#### 2.2.1. Standard Deviations of the System State Components

Output estimates are dependent on KF tuning; tuning “ration” computational attention between processes, measurement noise and *a priori* information. Through filter tuning, the performance can be evaluated by assessing its associated *a posteriori* covariance matrix ${\text{P}}_{k}^{+}$. This is demonstrated by the fact that any estimated system state tends to be bounded between one standard or two standard deviations (66% or 95% confidence, respectively). These standard deviation bounds are defined by the square roots of the corresponding diagonal elements in the computed ${\text{P}}_{k}^{+}$ matrix (standard deviation $\sigma_{k}\left(i\right)=\pm \sqrt{{\text{P}}_{k}^{+}\left(ii\right)}$). If an estimation error is exceeded by the *σ*- or 2*σ*-bound, then the performance of the KF under the parameter setting may have been set to unacceptable values.

#### 2.2.2. Test Statistics in the Domain of the Measurements

A global test of KF estimation could in theory be done by comparing predicted and true values of measurements. The measurement residual, called innovation, **d***_k_*, can be estimated and computed as a difference between the actual measurement, **z***_k_*, and the best available prediction based on the system model and previous measurement, ${\text{H}}_{k}\cdot {\hat{\text{x}}}_{k}^{-}$ [12]:

(5) $${\text{d}}_{k}={\text{z}}_{k}-{\text{H}}_{k}\cdot {\hat{\text{x}}}_{k}^{-}$$

where **H***_k_* is a measurement matrix and ${\hat{\text{x}}}_{k}^{-}$ is an *a priori* or predicted system state. The covariance matrix of innovation, **D***_k_*, is [12]:

(6) $${\text{D}}_{k}={\text{R}}_{k}+{\text{H}}_{k}\cdot {\text{P}}_{k}^{-}\cdot {\text{H}}_{k}^{T}$$

where **R***_k_* is a measurement noise covariance matrix and ${\text{P}}_{k}^{-}$ is a covariance matrix of any *a priori* system state. The innovation sequence is zero-mean, white (uncorrelated), with covariance equal to the measurement prediction covariance, and has a Gaussian distribution. The statistical test term in the domain of the measurements is defined by means of covariance matrix **D***_k_* and innovation vector **d***_k_* as normalized innovation squared (NIS) [1]:

(7) $$\Omega_{\text{d},k}^{2}={\text{d}}_{k}^{T}\cdot {\text{D}}_{k}^{-1}\cdot {\text{d}}_{k}$$

The null-hypothesis, which supposes that sample observations result purely from chance, is defined as:

(8) $$H_{0}:\;E({\text{d}}_{k})=0$$

and can be tested against an alternative hypothesis:

(9) $$H_{a}:\;E({\text{d}}_{k})\neq 0$$

The test statistic $\Omega_{\text{d},k}^{2}$ follows *χ*^2^-distribution with the probability relationship:

(10) $$P\{\Omega_{\text{d},k}^{2}\leq \chi_{m,1-\alpha }^{2}|H_{0}\}=1-\alpha$$

where *m* is the number of observations at epoch *t_k_* and corresponds to the degrees of freedom. *α* is a significance level. If $\Omega_{\text{d},k}^{2}<\chi_{m,1-\alpha }^{2}$, the null-hypothesis in Equation (8) can be accepted, and we can conclude that there is no significant discrepancy between a system estimate and a measurement model. If there is a significant innovation recognized, the null-hypothesis should be rejected. The causes of incompatibility must then be localized. The reasons for such a discrepancy could be either in the prediction, the stochastic model used in the KF or in the observations.

#### 2.2.3. Test Statistics in the System State Domain

In the system state domain, we test to see whether the KF-filtered system state is comparable to any previous knowledge we may have about the system. To address this comparison, the difference between the filtered ${\hat{\text{x}}}_{k}^{+}$ and predicted ${\hat{\text{x}}}_{k}^{-}$ value of the system state should be analyzed. The system state correction or residual **v**_x_,*_k_* can be written as:

(11) $${\text{v}}_{\text{x},k}={\hat{\text{x}}}_{k}^{+}-{\hat{\text{x}}}_{k}^{-}={\text{K}}_{k}\cdot {\text{d}}_{k}$$

Matrix **K***_k_* is a Kalman gain matrix that weights the measurement residuals. The system state residual represents the difference between the system state estimate before and after a measurement update. If this residual has a large value, it indicates that we are not predicting the future system state very well, because when new measurements are applied, there is a large jump in the state estimate. The corresponding covariance matrix, **P_V_***_x,k_*, is given by [12]:

(12) $${\text{P}}_{{\text{v}}_{x,k}}={\text{K}}_{k}\cdot {\text{D}}_{k}\cdot {\text{K}}_{k}^{T}$$

The system state corrections have a normal distribution **v***_x,k_* ∼ *N*(0, **P**_**V**_*_x,k_*), and the appropriate test statistics for the system state corrections is [12]:

(13) $$\Omega_{{\text{v}}_{x,k}}^{2}=\frac{{\text{v}}_{x,k}^{T}\cdot {\text{P}}_{{\text{v}}_{x,k}}^{-1}\cdot {\text{v}}_{x,k}}{\sigma_{0}^{2}}∼\chi_{n,1-\alpha }^{2}$$

where *n* is a number of unknowns in epoch *t_k_*. $\sigma_{0}^{2}$ is a reference variance.

## 3. Implementation and Numerical Analysis

The theoretical backgrounds given in Section 2 are demonstrated using a practical example.

### 3.1. Simulation of a Kinematic Process

**Figure 1.**
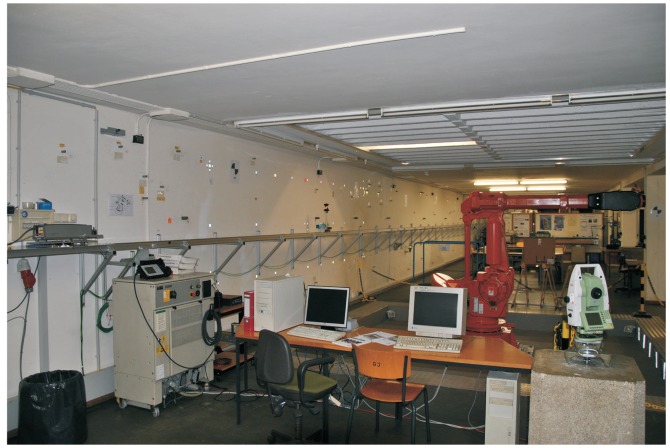
Geodetic Laboratory of the Chair of Geodesy at the Technical University of Munich [10]. © [2014] IEEE. Reprinted, with permission, from [The use of Kalman filtering in combination with an electronic tacheometer, Gamse, S.; Wunderlich, T.A.; Wasmeier, P.; Kogoj, D. IEEE Proceedings. 15-17 September 2010].

The kinematic observations were performed on a known reference trajectory at the Geodetic Laboratory of the Chair of Geodesy at the Technical University of Munich, Germany; Figure 1. The construction accuracy of the trajectory is 1 *mm* in both horizontal and vertical position. The “kinematic process” was performed as a tracking problem, with the electronic tacheometer, TCRA1201 Leica Geosystems, used to perform automatic measurements of a moving Leica Geosystems 360°-reflector, installed on a moving trolley. A more detailed description of experimental set-up can be found in [9,10].

#### 3.1.1. Kalman Filter Model for the Simulated Process

Because of the high frequency of measurements we obtained (*t_sample_* = Δ*t* = 0.125 *s*), the movement of the reflector can be described as a movement with approximately constant acceleration *a* during each sampling period of a length Δ*t*, which is equal to the time-stamp between any two measurement epochs. A discrete third-order kinematic model (discrete Wiener process acceleration model, DWPAM) [1], which is a derivation of KF, was used to process all of the captured measurement triples. The model is three-dimensional per coordinate. In our case, the observed and recorded measurements were horizontal angles *hz*, zenith distances *zr* and slope distances *d*, and the variables of interest are the position, velocity and acceleration components.

We rewrite main elements of a functional model. More details are given in [10]. The KF process equation, where no deterministic component is present, has the following form [10]:

(14) $${\text{x}}_{k+1}={\text{F}}_{k}\cdot {\text{x}}_{k}+{\text{Γ}}_{k}\cdot {\tilde{w}}_{k}$$

“where *w˜ _k_* is the uncorrelated white process noise and represents the acceleration increment during the *k*-th sampling period. The process noise enters into the system dynamics through the vector gain **Γ***_k_*:

(15) $${\text{Γ}}_{k}={\left[\begin{array}{ccccccccc} \frac{\Delta t^{2}}{2} & \Delta t & 1 & \frac{\Delta t^{2}}{2} & \Delta t & 1 & \frac{\Delta t^{2}}{2} & \Delta t & 1 \end{array}\right]}^{T}$$

The system state x*_k_* in our case is:

(16) $${\text{x}}_{k}={\left[\begin{array}{ccccccccc} x & \upsilon_{x} & a_{x} & y & \upsilon_{y} & a_{y} & z & \upsilon_{z} & a_{z} \end{array}\right]}_{k}^{T}$$

where *x, y, z* are the position components of the moving prism and *υ_x_,υ_y_, υ_z_* and *a_x_, a_y_, a_z_* are velocity and acceleration in all three directions, respectively. The transition matrix **F***_k_* in Equation (14) is:

(17) $${\text{F}}_{k}(\Delta t)=\left[\begin{array}{ccccccccc} 1 & \Delta t & \frac{{\left(\Delta t\right)}^{2}}{2} & 0 & 0 & 0 & 0 & 0 & 0\\ 0 & 1 & \Delta t & 0 & 0 & 0 & 0 & 0 & 0\\ 0 & 0 & 1 & 0 & 0 & 0 & 0 & 0 & 0\\ 0 & 0 & 0 & 1 & \Delta t & \frac{{\left(\Delta t\right)}^{2}}{2} & 0 & 0 & 0\\ 0 & 0 & 0 & 0 & 1 & \Delta t & 0 & 0 & 0\\ 0 & 0 & 0 & 0 & 0 & 1 & 0 & 0 & 0\\ 0 & 0 & 0 & 0 & 0 & 0 & 1 & \Delta t & \frac{{\left(\Delta t\right)}^{2}}{2}\\ 0 & 0 & 0 & 0 & 0 & 0 & 0 & 1 & \Delta t\\ 0 & 0 & 0 & 0 & 0 & 0 & 0 & 0 & 1 \end{array}\right]$$

and the process noise covariance matrix for one dimension ${\text{Q}}_{k}^{\left(1\right)}$is:

(18) $${\text{Q}}_{k}^{\left(1\right)}=\left[\begin{array}{ccc} \frac{\Delta t^{4}}{4} & \frac{\Delta t^{3}}{2} & \frac{\Delta t^{2}}{2}\\ \frac{\Delta t^{3}}{2} & \Delta t^{2} & \Delta t\\ \frac{\Delta t^{2}}{2} & \Delta t & 1 \end{array}\right]$$

The process noise covariance matrix for all three dimensions **Q***_k_* is:

(19) $${\text{Q}}_{k}=\sigma_{\omega }^{2}\cdot \left[\begin{array}{ccc} {\text{Q}}_{k}^{\left(1\right)} & 0 & 0\\ 0 & {\text{Q}}_{k}^{\left(1\right)} & 0\\ 0 & 0 & {\text{Q}}_{k}^{\left(1\right)} \end{array}\right]$$

where *σ_ω_* is a process noise intensity scalar” [10], p. 3.

In the KF measurement equation, the measurement vector **z***_k_* is composed of the reflector position components for each time step *t_k_, x, y* and *z*, which were computed out of the KF loop, from direct measurements of horizontal direction *hz_k_*, zenith distance *zr_k_* and slope distance *d_k_*, according to a defined right-handed coordinate system for each time step *t_k_* and given coordinates of the instrument stand point. The observation matrix **H***_k_* is [10]:

(20) $$\text{H}=\left[\begin{array}{ccccccccc} 1 & 0 & 0 & 0 & 0 & 0 & 0 & 0 & 0\\ 0 & 0 & 0 & 1 & 0 & 0 & 0 & 0 & 0\\ 0 & 0 & 0 & 0 & 0 & 0 & 1 & 0 & 0 \end{array}\right]$$

The law of propagation of variances and covariances should be used to calculate the covariance matrix of KF observations **z***_k_*, where the covariance matrix of direct measurements *hz, d* and *zr* is defined with standard deviations, *σ_hz_* = 1″, *σ_d_* = 5 mm, *σ_zr_* = 1″, given with the instrument technical specifications, and is constant through the process.

A more detailed mathematical description of the KF model for numerical experiment is given in [10].

### 3.2. Evaluation of the Developed Mathematical Model

**Figure 2.**
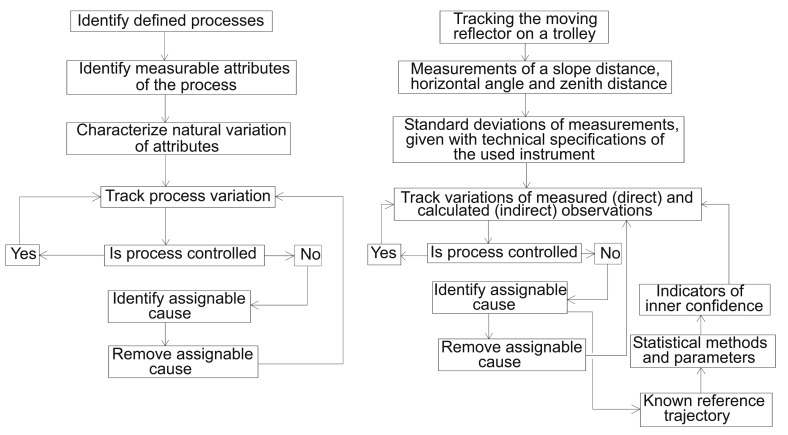
(**Left**) Statistical process control [11]. [2014] CSIAC. Reprinted, with permission, from [Statistical Process Control, Walker, E. https://sw.csiac.org/techs/abstract/347157. Report Date: 10 March 2004]; (**Right**) key steps for implementing the statistical process for the case of the simulated geodetic kinematic process.

In this subsection, we present the practical implementation of the theory behind controlling the kinematic process and evaluating output estimate values of the KF model. The performance of our model was evaluated using elements of inner confidence, statistical tests and an independent reference frame (Figure 2). We next define the errors present and determine control limits for the system state components of interest.

### 3.3. Testing KF DWPAM with Indicators of Inner Confidence

#### 3.3.1. Controllability of KF DWPAM

The controllability matrix **C** ∈ 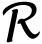
^9×9^ of the KF DWPAM was computed according to Equation (1), where instead of matrix **G**, matrix **Γ***_k_*, Equation (15), is used, because system dynamics are independent of any control signal, *i.e.*, **G** = 0, and process noise enters into the system dynamics through the matrix **Γ***_k_*. The rank of computed matrix **C** is then:

(21) rank(C)=3

which is not equal to the number of system state components, *n* = 9. At first sight, we might conclude that our model is not controllable. However, when we look closer at the structure of the matrix F and the matrix **Γ**, Equations (15) and (17), respectively, we can see equivalency in three dimensions. The multiplication of these two matrices leads to the controllability matrix **C**, whose rank is equal to three; *i.e.*, the number of system state components for each coordinate direction. We conclude that our KF DWPA model is controllable for all three components (position, velocity and acceleration) in all three dimensions.

#### 3.3.2. Observability of KF DWPAM

The observability matrix **O** ∈ 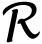
^27×9^ of the KF DWPAM model was computed according to Equation (2). Our model satisfies the observability condition, where the rank of the observability matrix is equal to the dimension, *n*, of the system state vector **x***_k_*:

(22) rank(O)=n=9

Condition Number of the Observability Matrix **O**

The condition number was computed according to Equation (3). This value measures the sensitivity of a solution of a linear system of equations to errors in observational data. Values for the conditional number near one indicate a well-conditioned matrix. For our KF model, the condition number of the observability matrix is constant through the process and its value is:

(23) cond(O)=28.132

The reciprocal of above condition number is:

(24) reciprocal_cond(O)=0.036

From the above two numerical values, we conclude that our model is observable.

#### 3.3.3. Determinant of the State Transition Matrix F*_k_*

The determinant of the state transition matrix of KF DWPAM is *det*(**F***_k_*) = 1, which means that the system of linear equations has a unique solution.

#### 3.3.4. Properties of the A *Posteriori* System State Covariance Matrix ${\text{P}}_{k}^{+}$

##### Trace of Matrix ${\text{P}}_{k}^{+}$

The condition of the convergence of the *a posteriori* system state covariance matrix ${\text{P}}_{k}^{+}$ is satisfied for our KF DWPAM (see [10], Figure 2).

##### Eigenvalues of Matrix ${\text{P}}_{k}^{+}$

The symmetry and positive definiteness of the covariance matrix **P***_k_* for our KF DWPAM were controlled using MATLAB operations.

##### Condition Number of Matrix ${\text{P}}_{k}^{+}$

**Figure 3.**
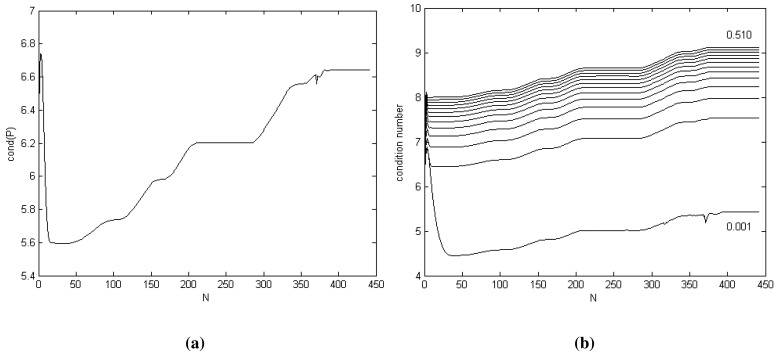
Condition number of matrix ${\text{P}}_{k}^{+}$. (**a**) $\kappa ({\text{P}}_{k}^{+})$, *σ_ω_* = 0:010; (**b**) $\kappa ({\text{P}}_{k}^{+})$, *σ_ω_*; *σ_ω_* = 0:001 : 0:040 : 0:51.

**Figure 4.**
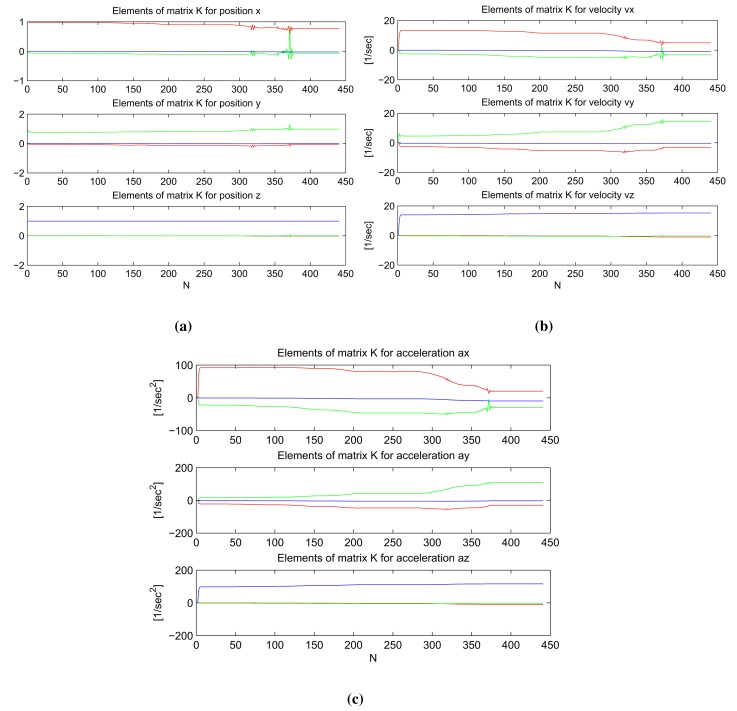
Elements of weighting matrix **K**. (**a**) Elements of weighting matrix **K**: position; (**b**) Elements of weighting matrix **K**: velocity. (**c**) Elements of weighting matrix **K**: acceleration.

**Figure 5.**
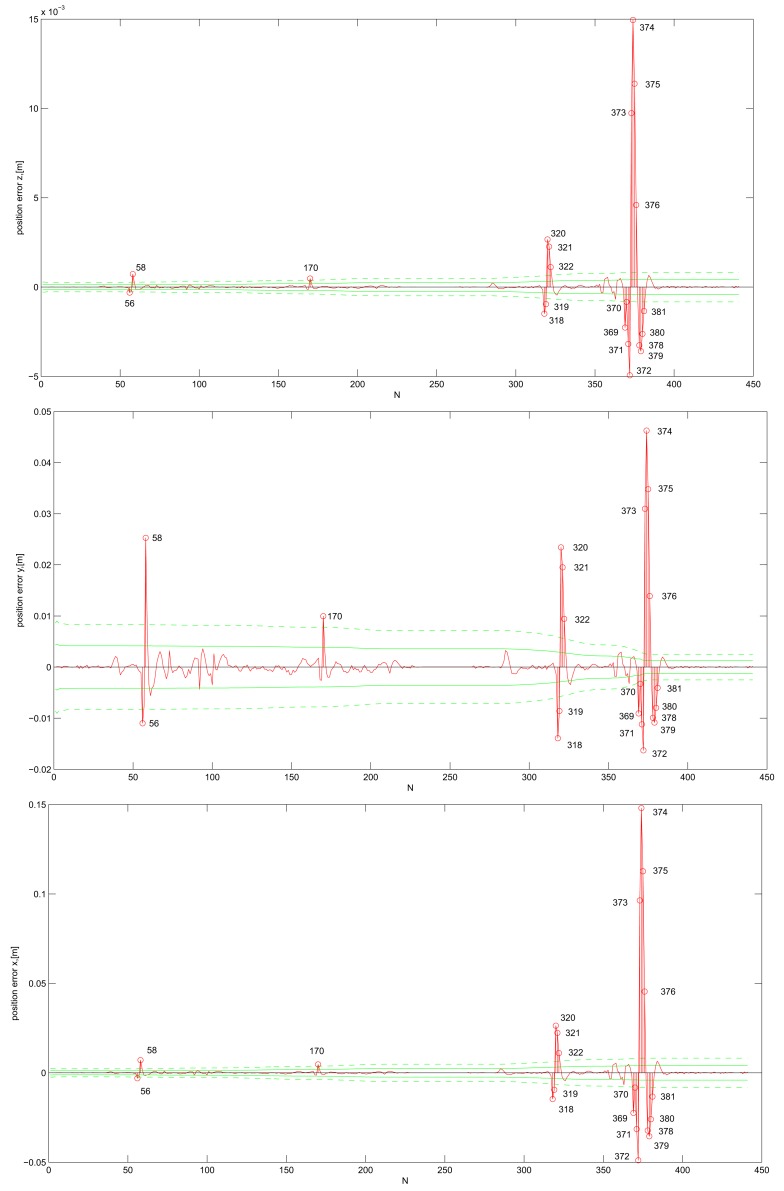
Position errors.

If we look at the standard deviations of the system state components, computed through the process (see [10], Figures 3, 4 and 5), we notice different ranks for the precision of position, velocity and acceleration. This is due to the different units on each parameter and because position components are better observed than velocity and acceleration.

The condition number of matrix ${\text{P}}_{k}^{+}$ for *σ_ω_* = 0.010 is shown in Figure 3a. A large condition number indicates near-singularity. For our KF DWPAM, we determine that the condition number is acceptable. Because the value of the condition number depends strongly on the value of the process noise intensity scalar *σ_ω_*, the $\kappa ({\text{P}}_{k}^{+})$ value was performed for different values of *σ_ω_, σ_ω_* = 0.001 : 0.040 : 0.51; Figure 3b. The lower graph represents the condition number for the lowest *σ_ω_, i.e., σ_ω_* = 0.001.

#### 3.3.5. Properties of the Kalman Gain Matrix K

In Figure 4, graphs of the elements of the weighting matrix K are plotted for the position, velocity and acceleration components in all three dimensions. In each graph, the red, green and blue lines represent the element of matrix K for the *x, y* and *z*-component of measurement correction. From these graphs, we conclude:

(1) The corresponding weight of KF measurement corrections, $\left({\text{z}}_{k}-{\text{H}}_{k}\cdot {\hat{\text{x}}}_{k}^{-}\right)$, is always the biggest for each component of the system state in the sense of coordinate direction. For example, the correction for position in *x* direction is:
  $${\text{corr}}_{x}={\text{K}}_{11}\cdot {\left({\text{z}}_{k}-{\text{H}}_{k}\cdot {\hat{\text{x}}}_{k}^{-}\right)}_{11}+{\text{K}}_{12}\cdot {\left({\text{z}}_{k}-{\text{H}}_{k}\cdot {\hat{\text{x}}}_{k}^{-}\right)}_{21}+{\text{K}}_{13}\cdot {\left({\text{z}}_{k}-{\text{H}}_{k}\cdot {\hat{\text{x}}}_{k}^{-}\right)}_{31}$$
  where the element **K**_11_, which incorporates *x* component of KF measurement correction, is the biggest; Figure 4a; elements of **K** for position *x*, red line.
(2) If we look at the graphs for elements of matrix **K**, which involves incorporating position corrections (Figure 4a), we see that for each position component, the weight of corresponding KF measurement correction in the same coordinate direction is about one, and for the other two KF measurement corrections in perpendicular directions, they are about zero. The same trends exist for velocity (Figure 4b) and acceleration (Figure 4c).
(3) When we compare weights for position—*x, y, z*—velocity—*υx, υy, υz*—and acceleration—*ax, ay, az*—we see that weights for position are of rank 10^0^, for velocity of rank 10^1^ and for acceleration of rank 10^2^. The reason for this is probably related to the different units of each of these system state components.

In our model, the values of the elements in matrix **K** are dependent on the covariance matrix of KF measurements, **R***_k_* (see [10], Equation (13)) and cannot be computed off-line. The elements of weighting matrix **K** depend very heavily on the value of process noise intensity scalar *σ_ω_*, specifically the elements that define velocity and acceleration corrections. We performed several tests with different values of *σ_ω_* to confirm numerically the mathematical validity of our KF model algorithm; *i.e.*, when the process noise is small, there is a high confidence in model and predicted values; therefore, the measurement corrections are weighted less heavily than in the case of big process noise.

### 3.4. Statistical Tests of KFDWPAM

Next, statistical tests in the domain of the measurements and system state were performed to explore possible reasons for the discrepancies we observed between estimation error and *σ*-bounds.

#### 3.4.1. Standard Deviations of the System State Components in Vector ${\hat{\text{x}}}_{k}^{+}$

The main intent of our simulation and KF model was to estimate the position of the moving reflector in three dimensions. For all three estimated position components, we observed a bigger dispersion of predicted and better fitting of filtered values when both are compared to the true/reference positions. The numerical results are given in Table 1.

Ideally, variances of the system state estimates should always include the true state within their errors. We visually present this test by plotting the standard deviations of the system state estimates, that is the square roots of the variances, with the estimation error plots. The standard deviations, computed with the KF, indicate how certain it is that the true state lies within a certain distance from the estimated state. The KF is about 66% (95%) certain that the true state element lies within the 1*σ* (2*σ*) confidence interval; *i.e.*, within one (two) deviation from the estimated element. If we plot the deviations into the error plot, ideally the error stays under the deviation lines. For KF DWPAM and *σ_ω_* = 0.1, the 1*σ*- and 2*σ*-bounds are drawn for each position component separately; Figure 5. Numerical values are given in Table 2. The number of steps, where the position error exceeds the 2*σ*-bound, is also plotted. In all three figures, the position error is represented with a red solid line, *σ*-bound with a green solid line and 2 · *σ*-bound with a dashed solid line. All plots represent cases where no gross errors (large outliers) were present and for *σ_ω_* = 0.1.

Table 2 shows the reduction of the condition number (at the same time, there is better convergence of trace ${\text{P}}_{k}^{+}$) with bigger confidence into the model (smaller *σ_ω_* value). However, at the same time, there is a big reduction of position error, where less true states lie inside the 1*σ*- or 2*σ*-bound of estimated positions, which means a high discrepancy between the estimated and true position system state components. For *σ_ω_* = 0.1, we deduce that position state estimates are consistent with their computed standard deviations and *σ*-bounds. Position errors for all three position components fall about 91% and 95.5% within 1*σ* and 2*σ*-uncertainty regions. For *σ_ω_* = 0.01, position state estimates are inconsistent with their computed standard deviations. Only approximately 46% and 61% of estimated positions lie within the 1*σ*- and 2*σ*-bound, respectively. We also observe that there is an increasing trend in the deviation in the different position state estimates variables, but still bounded.

#### 3.4.2. Consistency of the Model in the Domain of the Measurements

**Figure 6.**
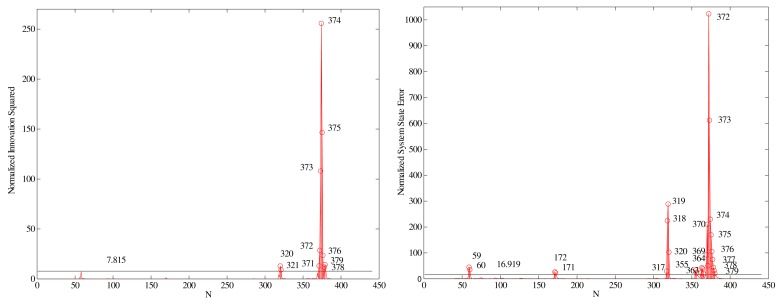
Normalized innovation squared (**Left**) and normalized system state error squared (**Right**), [10]. © [2014] IEEE. Reprinted, with permission, from [The use of Kalman filtering in combination with an electronic tacheometer, Gamse, S.; Wunderlich, T.A.; Wasmeier, P.; Kogoj, D. IEEE Proceedings. 15–17 September 2010].

We next test the KF measurement innovations together with their covariances using the normalized innovation squared (NIS), Equation (7), and the probability relationship given in Equation (10). For KF DWPAM, the value of degrees of freedom is equal *r* = 3, and a significance level value of *α* = 0.05 was used; *i.e.*, $P\{\Omega_{\text{d},k}^{2}\leq \chi_{3,1-0.05}^{2}|H_{0}\}=1-0.05$. At the 95% confidence level, for a measurement vector that includes three variables, the confidence region of the upper one-sided test is $\chi_{3,1-0.05}^{2}=7.815$. The normalized innovation squared for KF DWPAM is plotted in Figure 6, left.

The innovations represent measurement residuals, and for this analysis it is assumed that they are normal (Gaussian) distributed variables with zero mean and covariance, given in D*_k_*:

(25) $${\text{d}}_{k}∼N(0,{\text{D}}_{k})$$

**Figure 7.**
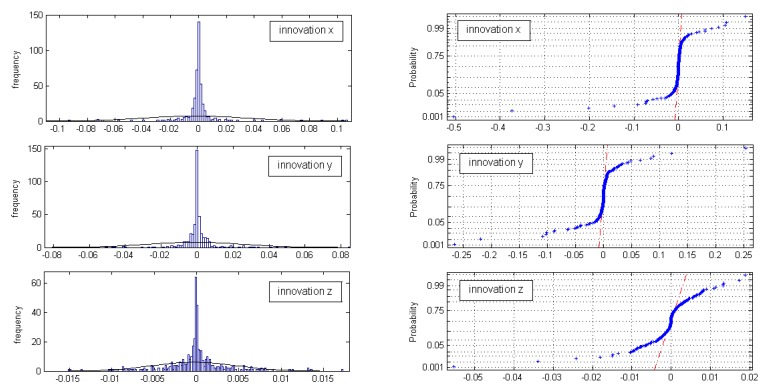
Probability distributions for innovations *x, y, z*: Histograms with a normal density function (**Left**) and normal probability plots (**Right**).

The normal probability plots for all three innovation sets (*x,y,z* innovation) do not show linear patterns on the whole data; Figure 7. In general, the data are plotted against a theoretical normal distribution in such a way that the points should form an approximate straight line. Departures from this straight line indicate departures from normality. Our data show a reasonably linear pattern in the center of the data, but the tails show departures from the fitted line. In our case, we must deal with short tail normal probability distributed data [13]. In the middle of these plots, the data show a weak S-like pattern. The first few and the last few data on each plot show marked departures from the reference fitted line. In the case of short tails, the first few points overestimate the fitted line above the line, and the last few points underestimate the fitted line. These tail-end outliers may indicate significant deviations from the assumed normal distribution of the measurement innovations.

In Table 3, the estimates of mean *μ* and the estimate of the standard deviation *σ* of the normal distribution for each innovation set are given at the 95% confidence interval.

#### 3.4.3. Test Statistics of the Model in the System State Domain

We next run a second formal test for the consistency of the model by examining the normalized system state errors. We ran single-run tests with different process noise scalars *σ_ω_* to check the KF DWPAM consistency in the domain of the system state. Using a process noise variance *σ_ω_* = 0.1, the normalized system state error squared is shown in Figure 6, right. The confidence region for a nine-degree-of-freedom system state vector (*n* = 9) is 16.919 at a 95% confidence level. KF estimation resulted in 26 points out of *N* = 441 to be outside the 2*σ* confidence region; this represents 6% of the total. The statistical test for testing model consistency in the system domain was computed using Equation (13), with the *a priori* variance computed using a preliminary adjustment and its value set accordingly to $\sigma_{0}^{2}=0.01$.

## 4. Using the Reference Trajectory as an Additional Estimator Parameter

**Figure 8.**
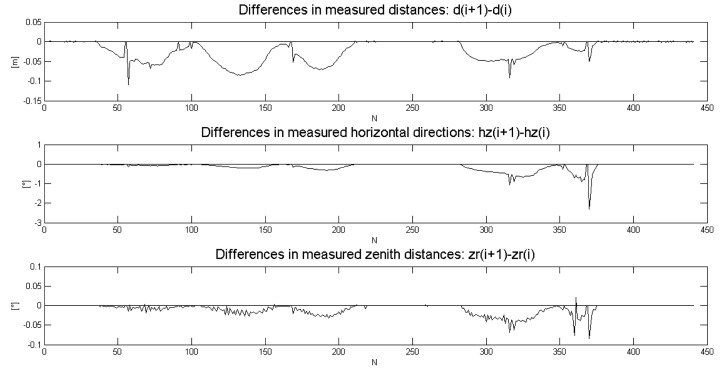
Direct measurements.

**Figure 9.**
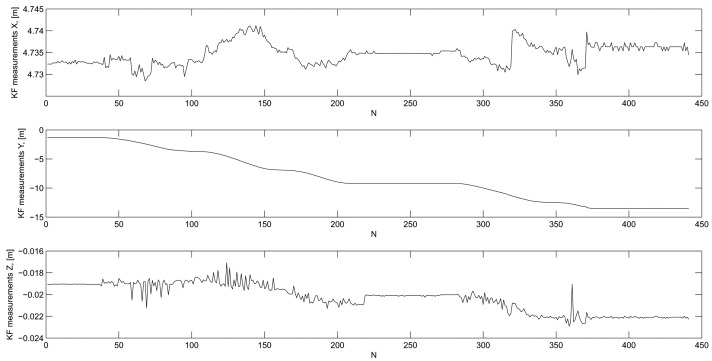
Indirect or KF measurements.

The most direct way to evaluate KF DWPAM accuracy and to find reasons for discrepancies is to use data of direct measurements of distance, horizontal angle and zenith distance and indirect measurements of trolley position on a known reference trajectory In Figure 8, the differences between successive measurement steps *k*(*i* + 1) − *k*(*i*) of the direct measurements are plotted. The rapid changes in measurements coincide with the observed discrepancies in statistical tests of the model that were discussed above. The reason for these deviations could thus lie in model, in direct observations or in the true motion of the trolley. True trolley motion might have included jerky changes in the inclination of the reflector. This is particularly possible in the *x*-direction, perpendicular to the trajectory. From our observed changes in direct (KF) measurements (Figure 8) and indirect measurements (Figure 9), we conclude that the reason for the KF DWPAM inconsistency lies in the actual discontinuous motion of the trolley. The KF model cannot follow rapid changes in motion very quickly; consequently, when fast changes occur, bigger values of differences between predicted and true measurement also occur, as in the case of continuous motion.

In our practical example, we assumed and then tested the Gaussian distribution of innovations. We also reason that through the computation of the indirect (KF) measurements from direct measurements, a non-Gaussian distribution can occur. Our tests (default null hypothesis) also confirm that only sample innovations where no big changes in measurements occur have a Gaussian distribution.

## 5. Conclusions

In this contribution, we presented the theoretical background and a practical example of using and testing a Kalman filter model to control simple trajectory motion. In previous works [9,10], the well-known statistical tests in the system state domain and in the domain of the measurements were presented.

The main contribution of this work is to perform additional parameters for the consistency check of the KF model. The mathematical background and numerical computation for the simulated example are given.

(1) First, the determinant of the state transition matrix is checked. It has a non-zero value in a practical example, which confirms that the system has a unique solution.
(2) The observability and controllability are checked with the values of their matrix rank and with more quantitative measure, given as the condition number of observability matrix. The rank of the observability matrix is equal to the number of unknown components, *i.e.*, the dimension of the system state vector. Furthermore, the value of the observability matrix condition number is acceptable for our model. The rank of the controllability matrix is equal to three, which is the number of system state components in each direction. The rank is not equal to the total number of the system states. The reason can lie in the pre-assumption that the unknowns in each direction are uncorrelated from each other, which influences the structure of the state transition matrix.
(3) Beside a well-known condition of the convergence of the trace, additional properties of the *a posteriori* system state covariance matrix are observed. The condition number of the *a posteriori* system state covariance matrix was computed for different values of the process noise intensity scalar, where the values show convergence to an acceptable maximum.
(4) In the contribution, the elements of Kalman gain matrix, which defines the weights of KF measurements, were observed. For each position is the weight of corresponding KF measurement correction in the same direction, about one, and for the other two KF measurement corrections in perpendicular directions, about zero. The reason can lie in the pre-assumption that the unknowns in each direction are uncorrelated from each other.
(5) The performance of the normal distribution of measurement innovations enables the identification of the presence of the outliers. Errors in the measurement domain are generally not normally distributed. In real-time kinematic time series analysis, we may not have the ability to analyze the detailed nature of measurement errors with classical geodetic least squares adjustment, because for each time step, we will have only at least required a number of measurements. In the case of KF model, the distribution test is done on KF measurement innovations. KF measurement innovations, which represent measurement corrections, require a generally Gaussian distribution. With non-Gaussian errors, Kalman filtering does not produce an optimal general solution of a system state, but rather only the optimal solution for the linear system chosen. Therefore, special attention is required when reporting and using KF-derived confidence bounds. KF-derived variances should be accepted only stringently as representing measurement accuracy. It is then the task of the engineer to scale up or down uncertainty bounds for selected value using the process noise intensity scalar. In other words, the KF model can result in under- or over-estimated values and associated errors.

The highly accurate reference trajectory and the used precise measurement instrument offer the possibility to additionally test Kalman filter modeling and especially to find the reasons for discrepancies and offsets. By investigating direct measurements and differences between sequential measurement steps, conclusions about the reasons for big deviations between predicted and estimated system state components and big offsets in measurement innovations were made. The main reason lies in the jerky changes in the inclination of the reflector on the moving trolley.

Our practical example showed that statistical tests in the measurement domain and in the system state domain and other parameters of the statistical process control can provide appropriate tests for detecting discrepancies in the kinematic processes when no independent external control is available.

Extensions and improvements of the work presented could be gained by using particle or unscented filters [14] and better algorithms based on centroid weighted Kalman filters [15], which should better optimize prediction performance and correction iterations for the systems with non-Gaussian noise. To overcome the problem of incorporating large measurement innovations, adaptive KF with predefined sliding windows should be tested as a possible improved way to deal with the online elimination of outliers.

## Figures and Tables

**Table 1.** Sum of differences between predicted/filtered and measured values.

	**∑ |Predicted-Measured|**	**∑ |Filtered-Measured|**
	**[m]**	**[m]**
*x*	3.785	0.935
*y*	3.590	0.597
*z*	1.031	0.095

**Table 2.** Percentage of filtered positions inside the 1*σ*- and 2*σ*-bound and the condition number of matrix $\left({\text{P}}_{k}^{+}\right)$.

	**1*σ*-Bound (Approximately 66% Inside)**			**2*σ*-Bound (Approximately 95% Inside)**		

	*x* % inside	*y* % inside	*z* % inside	*x* % inside	*y* % inside	*z* % inside
*σ_ω_* = 0.10	$\kappa ({\text{P}}_{k}^{+})\to 8.115$, no gross errors					

	91.4	91.8	92.1	95.5	95.5	95.5

*σ_ω_* = 0.06	$\kappa ({\text{P}}_{k}^{+})\to 7.782$, no gross errors					

	87.3	87.3	87.8	92.1	92.1	92.1

*σ_ω_* = 0.01	$\kappa ({\text{P}}_{k}^{+})\to 6.642$, no gross errors					

	45.1	46.5	46.3	61.0	60.3	60.8

**Table 3.** Parameters of the normal distribution for innovations.

	*μ*[*m*]	**Confidence Interval** [*μ*][*m*]	*σ*[*m*]	**Confidence Interval** [*σ*][*m*]
innovation *x*	−0.0026	[−0.0059, 0.0008]	0.0360	[0.0338, 0.0385]
innovation *y*	−0.0011	[−0.0036, 0.0013]	0.0265	[0.0248, 0.0283]
innovation *z*	−0.0003	[−0.0007, 0.0002]	0.0049	[0.0046, 0.0053]
